# Supplementary material for: Fluid pressurisation and earthquake propagation in the Hikurangi subduction zone
Source: Nat Commun. 2021 Apr 30;12:2481. doi: 10.1038/s41467-021-22805-w (PMC8087711; doi:10.1038/s41467-021-22805-w)
Supplement: Supplementary file 1 — Supplementary Information [file 41467_2021_22805_MOESM1_ESM.pdf]

Supplementary material
Fluid pressurization and earthquake propagation in the Hikurangi subduction zone

Aretusini S., et al.

March 30, 2021

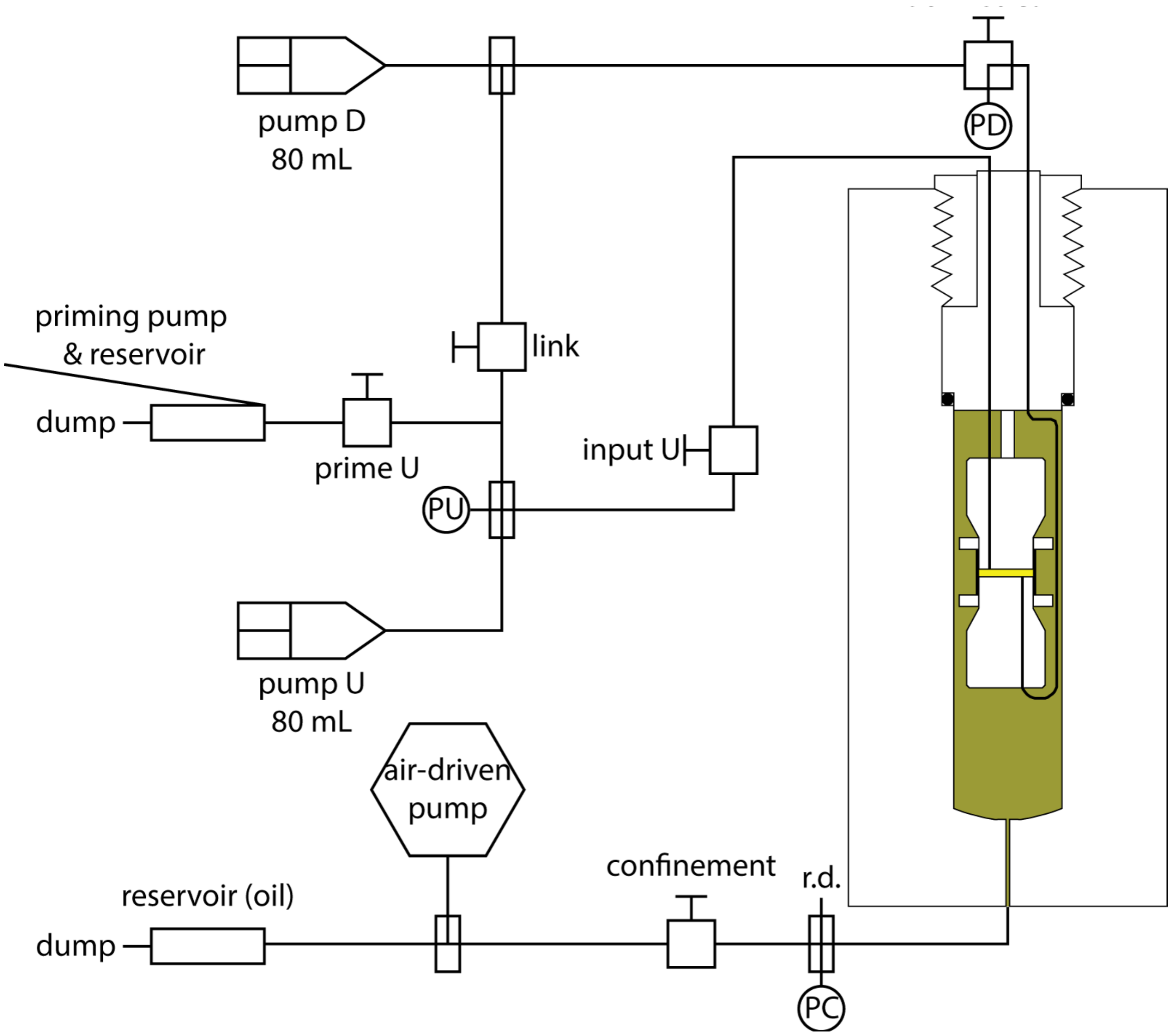

Supplementary Figure 1: The permeability measurement was performed in a permeameter under hydrostatic pressure conditions. Confining pressure was applied with an oil medium using an air-driven pump and was measured with a pressure transducer (PC). The pore fluid pressure was imposed with two independent syringe pumps, one for the upstream circuit (pump U) and one for the downstream circuit (pump D) and controlled with two independent pressure transducers (PU and PD, respectively). During the pore fluid oscillations applied in the upstream side, the link and downstream valves were closed to apply undrained conditions on the downstream side of the sample. The pressure vessel sketch was modified after Vinciguerra et al., (2005).

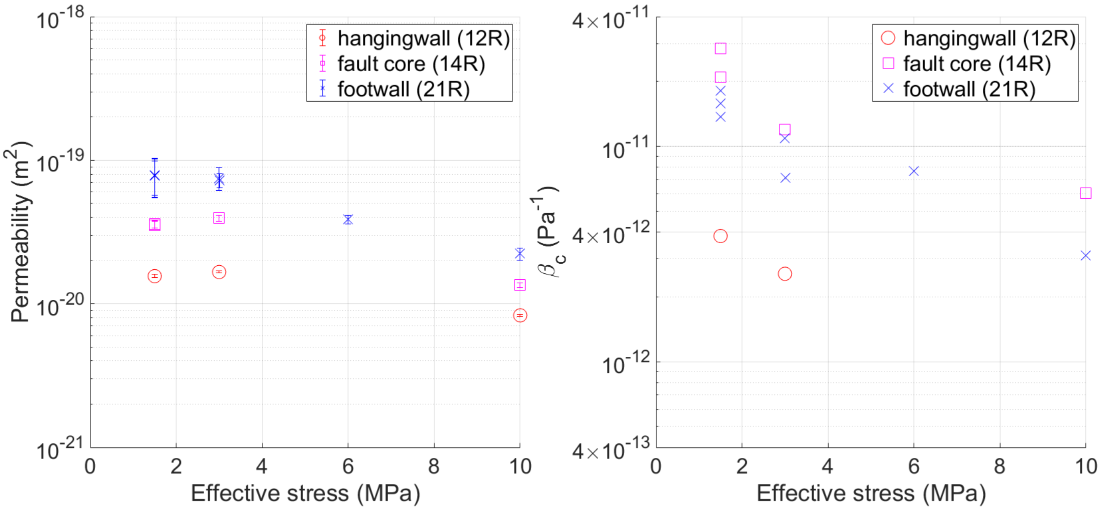

Supplementary Figure 2: Permeability (error bars indicate standard deviation) and pore compressibility of the fault core and host rocks (0-10 MPa).

P_c MPa	P_f MPa	P_{eff} MPa	k m2	s. d. m2	β 1/Pa	s. d. 1/Pa	η	s. d.	ξ	s. d.	gain	phase rad	period s
**12R1 (hangingwall)**, initial thickness: 4.41 mm, final thickness: 2.98 mm													
4.5	3	1.5	1.56e-20	4.31e-22	3.83e-12	6.36e-12	0.696	0.0172	0.0341	0.0566	0.325	1.26	120
6	3	3	1.67e-20	2.47e-22	2.56e-12	2.48e-12	0.744	0.00598	0.0228	0.0221	0.346	1.23	120
13	3	10	8.32e-21	2.3e-22	7.56e-12	4.40e-12	0.364	0.0091	0.0686	0.04	0.175	1.46	120
23	3	20	3.12e-21	2.59e-22	5.65e-12	1.29e-12	0.139	0.0114	0.0503	0.0115	0.068	1.62	120
33	3	30	1.91e-21	2.33e-22	4.31e-12	3.94e-12	0.17	0.0207	0.0384	0.0351	0.084	1.56	240
43	3	40	9.03e-22	5.06e-23	3.76e-13	1.867e-12	0.198	0.0108	0.0342	0.017	0.0972	1.53	600
63	3	60	7.32e-22	2.52e-23	1.31e-11	2.98e-12	0.245	0.00788	0.116	0.0265	0.117	1.61	900
103	3	100	5.03e-22	2.43e-23	1.58e-11	1.98e-12	0.18	0.00841	0.141	0.0176	0.084	1.74	960
**14R1A (fault core)**, initial thickness: 2.7 mm, final thickness: 1.97 mm													
4.5	3	1.5	3.53e-20	2.27e-21	2.85e-11	2.35e-11	0.585	0.0368	0.171	0.141	0.266	1.4	30
4.5	3	1.5	3.58e-20	2.22e-21	2.1e-11	1.65e-11	0.395	0.024	0.126	0.0992	0.186	1.49	20
6	3	3	3.95e-20	1.98e-21	1.2e-11	1.45e-11	0.435	0.0211	0.072	0.087	0.208	1.42	20
13	3	10	1.35e-20	5.08e-22	6.06e-12	1.15e-11	0.448	0.0158	0.0364	0.0689	0.216	1.38	60
23	3	20	5.8e-21	1.19e-22	1.94e-13	1.99e-12	0.384	0.00609	0.00116	0.0119	0.189	1.38	120
43	3	40	2.66e-21	2.09e-22	1.1e-11	1.33e-11	0.352	0.0272	0.0661	0.0798	0.169	1.46	240
63	3	60	1.83e-21	5.89e-23	2.4e-12	4.46e-12	0.399	0.0132	0.0144	0.0268	0.179	1.405	360
83	3	80	1.32e-21	4.35e-23	6.81e-12	9.31e-12	0.524	0.0159	0.0409	0.0559	0.25	1.34	720
103	3	100	8.36e-22	2.98e-23	4.07e-12	6.13e-12	0.399	0.0132	0.0245	0.0368	0.194	1.4	900
**21R3 (footwall)**, initial thickness: 2.16 mm, final thickness: 1.54 mm													
4.5	3	1.5	7.79e-20	2.07e-20	1.81e-11	5.17e-11	0.329	0.0876	0.0851	0.243	0.158	1.5	5.99
4.5	3	1.5	7.85e-20	2.34e-20	1.58e-11	1.45e-10	0.665	0.198	0.0742	0.68	0.308	1.29	12
4.5	3	1.5	7.88e-20	2.38e-20	1.37e-11	1.07e-10	0.501	0.151	0.0641	0.5	0.238	1.37	9
6	3	3	7.25e-20	8.21e-21	7.16e-12	4.13e-11	0.614	0.069	0.0336	0.194	0.29	1.29	12
6	3	3	7.48e-20	1.35e-20	1.09e-11	6.24e-11	0.476	0.0854	0.0512	0.293	0.228	1.38	9.01
9	3	6	3.87e-20	2.55e-21	7.65e-12	2.97e-11	0.492	0.0317	0.0359	0.14	0.236	1.36	18
13	3	10	2.23e-20	2.11e-21	3.11e-12	3.43e-11	0.472	0.0442	0.0146	0.161	0.229	1.35	30
23	3	20	9.47e-21	5.59e-22	4.85e-12	1.98e-11	0.401	0.023	0.0227	0.093	0.195	1.39	60
23	3	20	8.75e-21	7.02e-22	2.08e-12	2.51e-11	0.741	0.0586	0.00976	0.118	0.356	1.2	120
43	3	40	4.51e-21	2.55e-22	2.39e-11	1.47e-11	0.382	0.0209	0.112	0.0688	0.181	1.49	120
63	3	60	3.23e-21	1.24e-22	7.23e-12	1.02e-11	0.546	0.0196	0.0339	0.048	0.261	1.33	240
83	3	80	2.45e-21	2.52e-22	1.12e-11	3.61e-11	0.519	0.0529	0.0525	0.169	0.247	1.35	300
103	3	100	1.83e-21	9.2e-23	4.41e-14	1.15e-11	0.387	0.0187	0.000207	0.0541	0.19	1.37	300

Supplementary Table 1: P_c: confining pressure, P_f: pore fluid pressure, P_{eff}: effective pressure, k: permeability, β: undrained compressibility, η: dimensionless permeability, ξ: dimensionless storativity, gain: amplitude ratio of downstream over upstream waveform, phase: phase between downstream and upstream waveform, period: period of downstream and upstream waveforms. Standard deviations are indicated as "s. d." in the columns on the right of the respective variable.

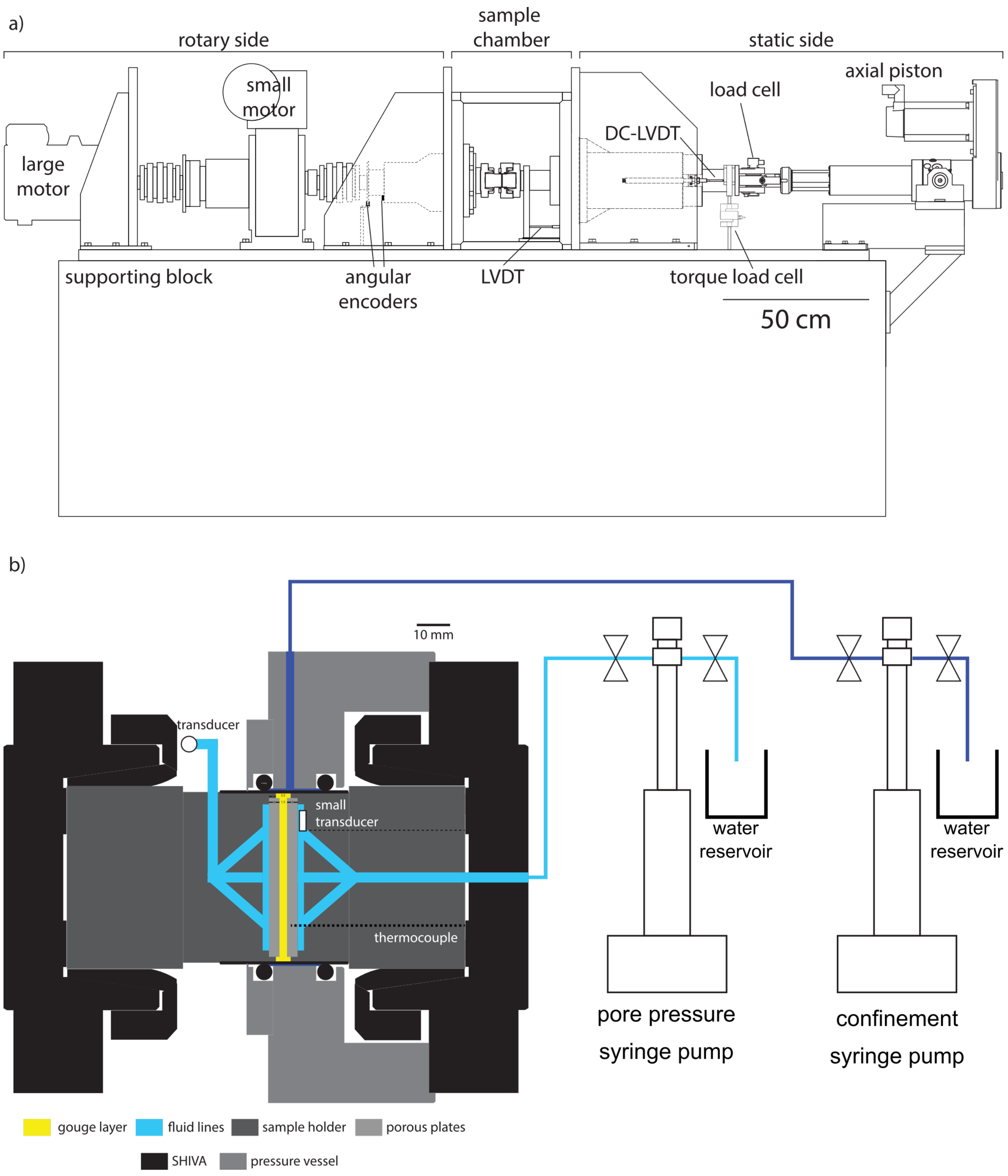

Supplementary Figure 3: a) Overview of the SHIVA apparatus with indicated the two electric motors, the axial piston system and the sensors described in the Methods. b) Magnified view of the sample chamber with indicated: gouge layer, sample holders, pore fluid lines and relative pore fluid and confining fluid circuits, upstream and downstream transducers and K-type thermocouple. Two syringe pumps were used to control pressure in the fluid circuits (not to scale).

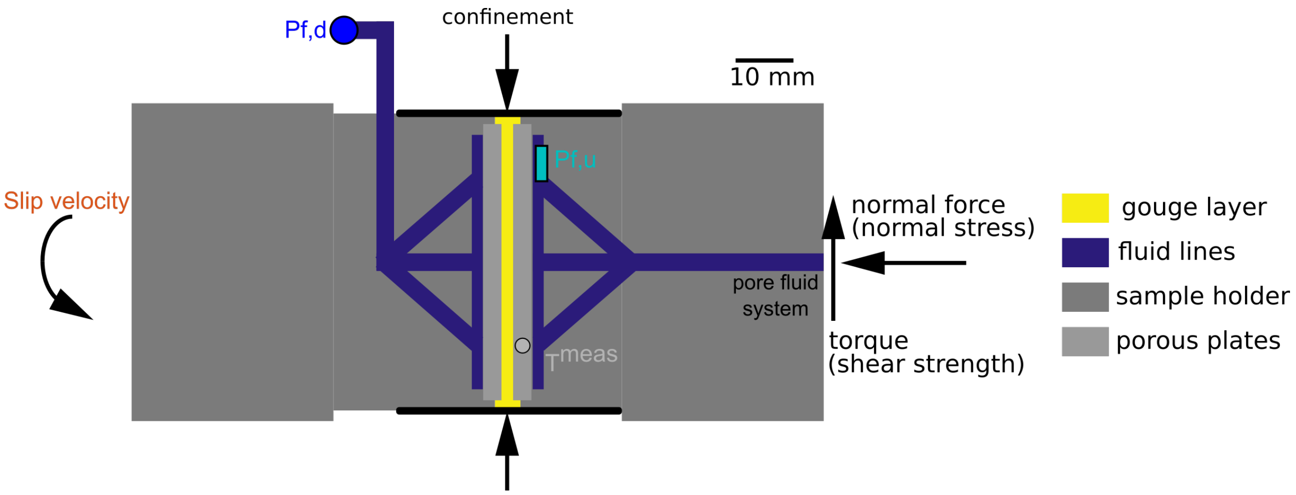

Supplementary Figure 4: Simplified view of Figure 3b. Boundary conditions of the experiments are represented as applied slip velocity and normal force, confining and pore pressure. Position of the pressure transducers (Pf, u and Pf, d) and of the thermocouple hot junction (T^{meas}) are indicated.

name	mat.	cond.	τ_p MPa	τ_{ss} MPa	$D_{w,t}$ m	α_t	R_t	W_b MJ/m^2	Δt_{dil} s	ΔP^d_{dil} MPa	ΔP^u_{dil} MPa	Δh_{dil} μm	Δt s	ΔP MPa	Δh μm	T^{mod}_{max} $°C$
s1736	HW	FP	2.11	0.27	0.17	0.52	0.97	0.079	0.1	-0.44	0	-6	50	0.09	66	60.2
s1737	FW	FP	2.62	0.42	0.38	0.42	0.88	0.128	0.08	-0.41	-0.45	0	29	1.07	109	82.6
s1738	FC	FP	2.06	0.21	0.08	0.57	0.96	0.056	0	0	-0.29	0	19	1.23	131	36.8
s1739	HW	FP	2.14	0.19	0.23	0.37	0.90	0.078	0.13	-0.68	-0.7	-8	21	0.94	268	56.3
s1741	FC	FP	1.94	0.55	0.12	0.53	0.97	0.047	0.05	-0.58	-0.13	-10	64	0.79	137	63.4
s1742	FW	FP	2.23	0.15	0.18	0.42	0.94	0.070	0.06	-0.44	-0.3	-6	20	1.23	80	53.5
s1823	C	FP	4.62	1.32	0.11	0.66	0.94	0.11	0.08	-1.87	-0.45	-69	0.27	0.99	186	490.2

(a) Fluid pressurised experiments.

name	mat.	cond.	τ_p MPa	τ_{ss} MPa	$D_{w,t}$ m	α_t	R_t	W_b MJ/m^2	Δh 10^{-6} m	T^{mod}_{max} $°C$
s1819	FC	WD	2.27	0.74	0.40	0.48	0.94	0.079	19	145.7
s1816	FC	WD	2.00	0.75	0.11	0.81	0.90	0.040	59	128
s1815	FC	RH	2.59	1.67	0.36	1	0.95	0.106	254	280.6
s1811	FC	RH	2.67	1.68	0.25	1	0.96	0.110	228	272.4

(b) Water dampened and room humidity experiments.

Supplementary Table 2: exp.: experiment name, material: provenance of tested materials: foot wall (FW), hanging wall (HW), fault core (FC), and Carrara marble gouge (C), experimental condition: fluid pressurized (FP), water dampened (WD), or room humidity (RH), τ_p: peak strength, τ_r: residual strength, $D_{w,t}$: slip weakening distance, α_t: exponent of the power law, R_t: weighted xi squared parameter (all obtained by the power law best fit procedure), W_b: breakdown work, Δt: delay of the pore pressure peak with respect to the beginning of the seismic slip velocity pulse, ΔP: amount of pore pressure increase with respect to the starting pore pressure (3 MPa), Δh: amount of thickness reduction during the seismic slip, Δt_{dil}, ΔP^u_{dil}, ΔP^d_{dil}, and Δh_{dil} are the corresponding quantities for the dilatant stage at the onset of slip; T^{mod}_{max} the maximum estimated temperature at the center of the gouge layer during the seismic slip velocity pulse (Methods).

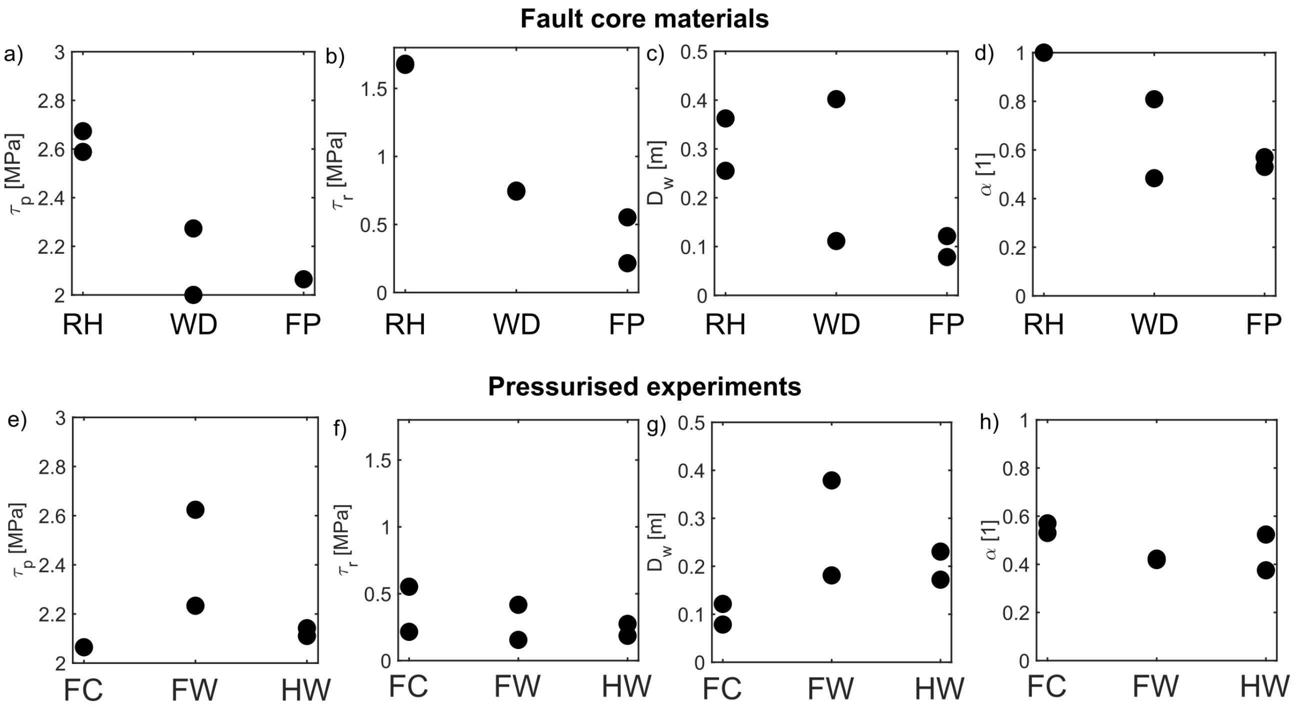

Supplementary Figure 5: Best fit parameters obtained from the seismic slip velocity pulse. From a) to b), the peak strength τ_p, residual strength τ_r, slip weakening distance D_w and exponent α obtained by fitting the experimental data with a power law function are grouped by the experimental conditions (room humidity RH, water dampened WD, and fluid pressurised FP), considering only the fault core starting material (FC). From e) to h), the peak strength τ_p, residual strength τ_r, slip weakening distance D_w and exponent α obtained by fitting the experimental data with a power law function are grouped by the starting material (fault core FC, foot wall FW, and hanging wall HW), considering only the fluid pressurised experimental conditions (FP).

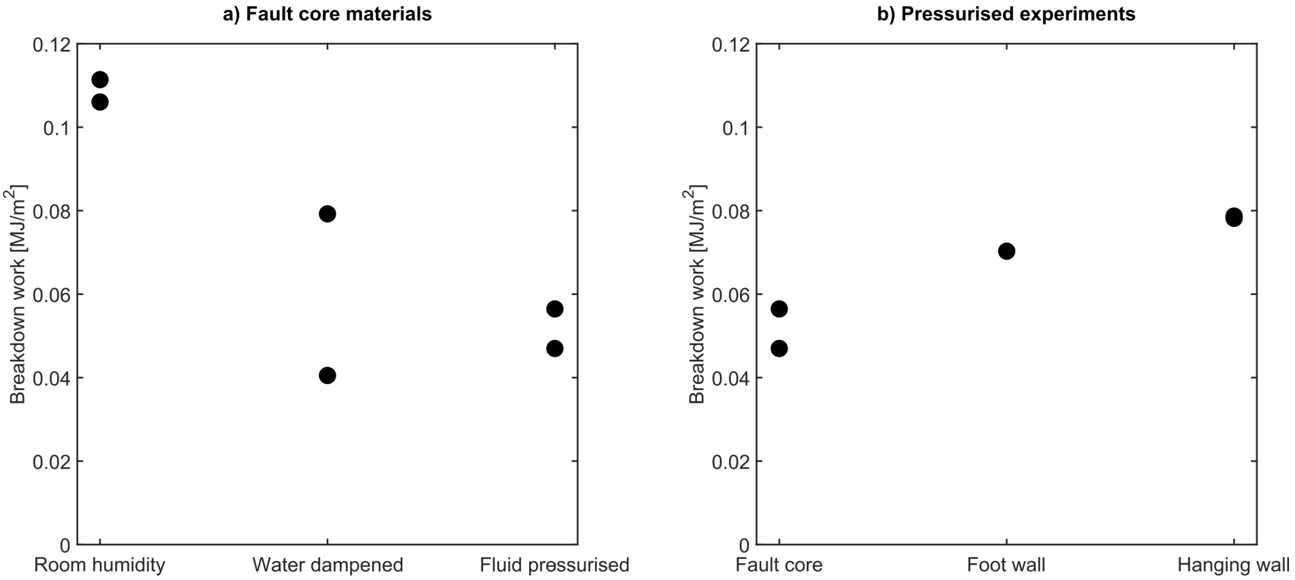

Supplementary Figure 6: Breakdown work values estimated from the seismic slip velocity pulse. a) Breakdown work obtained from the seismic slip velocity pulse for the fault core starting material (FC) with changing experimental conditions (RH, WD, FP). b) Breakdown work obtained from the seismic slip velocity pulse for all the starting materials (FC, HW, FW) under fluid pressurised experimental conditions (FP).

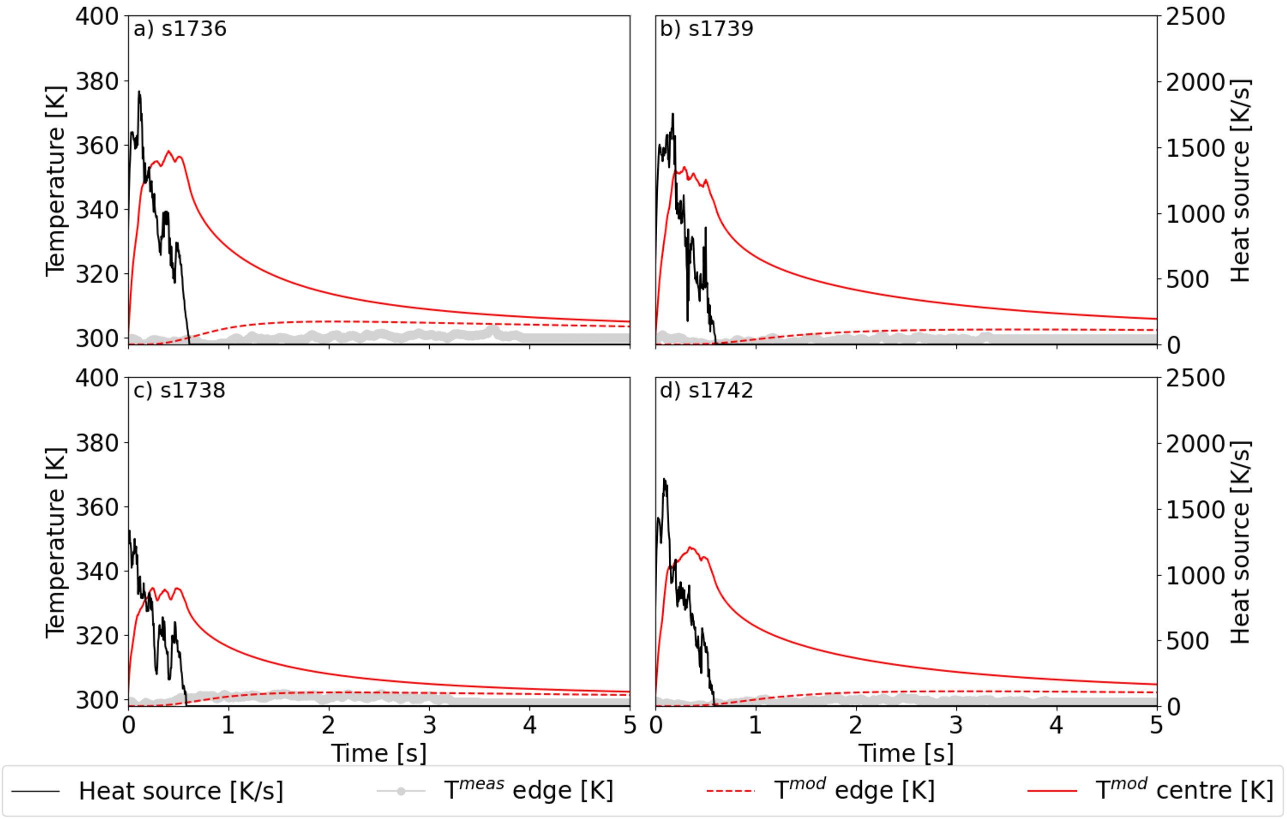

Supplementary Figure 7: Measured and estimated temperature in the fluid pressurized experiments. The temperature measured with the thermocouple ($T^{meas}edge$), was reproduced by the estimated temperature ca. 1 mm away from the gouge layer ($T^{mod}edge$). The maximum value of the estimated temperature at the centre of the gouge layer ($T^{mod}centre$) is reported in Table 2 as T^{mod}_{max}.

.

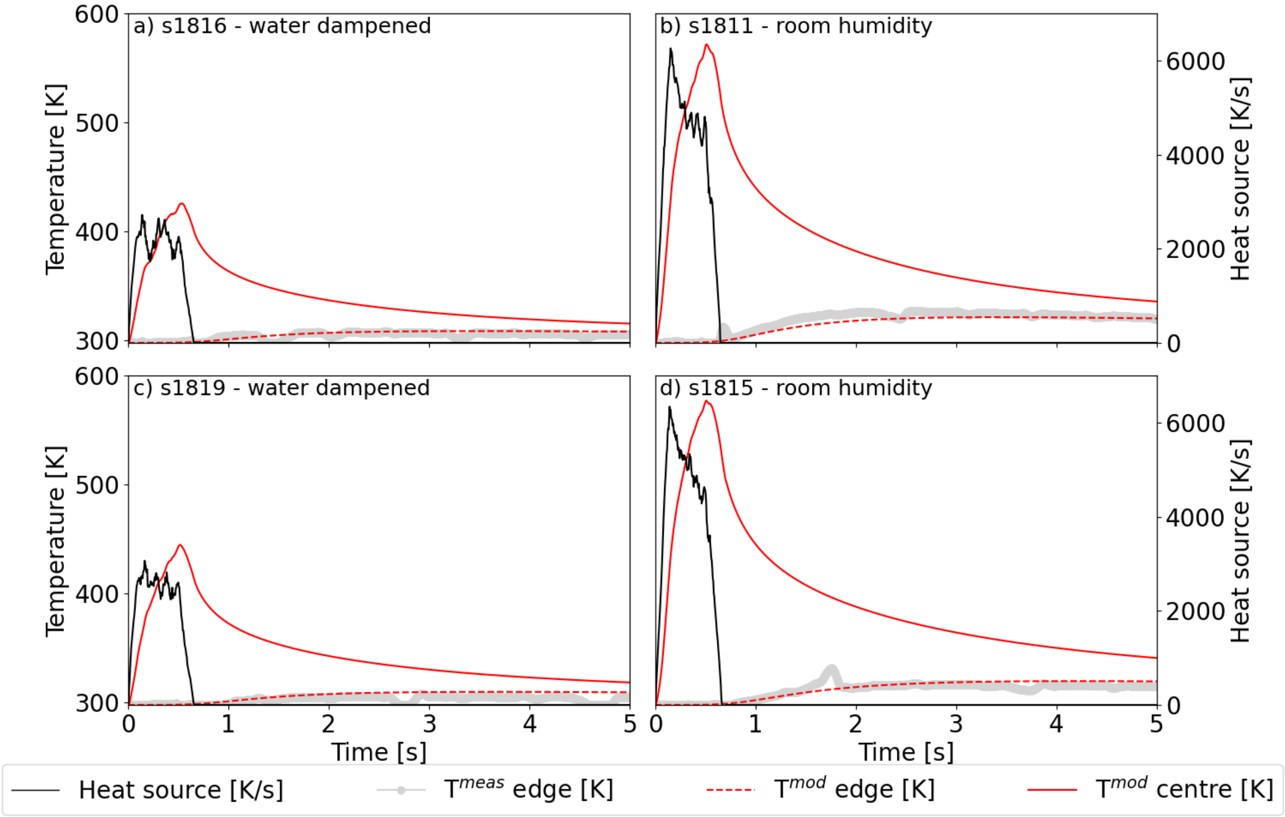

Supplementary Figure 8: Measured and estimated temperature in the water dampened and room humidity experiments. The temperature measured with the thermocouple ($T^{meas}edge$), was reproduced by the estimated temperature ca. 1 mm away from the gouge layer ($T^{mod}edge$). The maximum value of the estimated temperature at the centre of the gouge layer ($T^{mod}centre$) is reported in Table 2 as T^{mod}_{max}.
